# Current situation regarding psychedelics and magic mushroom in Korea

**DOI:** 10.1192/j.eurpsy.2024.1695

**Published:** 2024-08-27

**Authors:** J. S. Seo

**Affiliations:** Psychiatry, Gwangmyeong Hospital, Chung-Ang University, Korea, Gwangmyeong-si, Korea, Republic Of

## Abstract

**Introduction:**

Recently, the pros and cons have been debating in Korea even before the approval of use of medical marijuana with very strict limitations. And the next controversial topic is psychedelics. In 1890, when mescaline was first isolated from peyote cactus, clinical researches began, but due to its harmful effects, it was thereafter legally prohibited in 1970 in USA. However, a pernicious debate over the medical efficacy of psychedelic drugs has begun again with the release of a study that uses psychedelic mushrooms to be effective against treatment-resistant depression, alcohol dependence, and depression and anxiety in terminal cancer patient.

**Objectives:**

To make a consensus on the medical use of these, we reviewed wild mushrooms containing hallucinogenic ingredients living in Korea.

**Methods:**

To make a consensus on the medical use of these, we reviewed wild mushrooms containing hallucinogenic ingredients living in Korea.

**Results:**

Mushrooms have long been popular as a food ingredient in Korea. Psilocybin, a classical psychedelic, can be obtained from magic mushroom (Psilocybe cubensis). The psilocybin on the CNS and causes hallucinations. Intoxication symptoms include pleasant or nervousness, sudden laughter, hallucinations, visual impairment, tachycardia and hypertension, reflexes, agitation, cognitive impairment, confusion, and aggressive behavior. These symptoms last for 2-4 hours after ingestion, and most disappear within six hours.

Among 114 species of Psilocybe containing psilocybin around the world, only five wild mushrooms found in Korea that cause nervous system hallucinations are as follows: P. argentipes, P. coprophila, P. perdaria, and P. subcarulipes.

In Korea, there is acute poisoning case suffering with GI symptoms caused by mushrooms, but it is difficult to find records of abuse or dependences case caused by psychedelic mushrooms. In addition, although oriental medicine treatment is relatively active, it is not used as an herbal medicine.

**Conclusions:**

Currently, the Korean government classifies psychedelic mushroom-derived substances, Psilocybin and Psilocin, as psychotropic drugs by law. If researcher intends to clinical trial with eve very small amount of it for academic purpose, it is only possible after obtaining approval from Korean FDA. In order to determine the usefulness of psychedelics, many clinical studies are needed in Korea.

**Disclosure of Interest:**

None Declared

